# Modulation of the Fungal-Host Interaction by the Intra-Species Diversity of *C. albicans*

**DOI:** 10.3390/pathogens7010011

**Published:** 2018-01-17

**Authors:** Christina Braunsdorf, Salomé LeibundGut-Landmann

**Affiliations:** Section of Immunology, Vetsuisse Faculty, University of Zürich, Winterthurerstrasse 266a, CH-8057 Zürich, Switzerland; christina.braunsdorf@uzh.ch

**Keywords:** *Candida albicans*, intraspecies diversity, genomic and genetic variations, morphotypes, phenotypes, host-pathogen interaction

## Abstract

The incidence of human infections caused by the opportunistic fungal pathogen *Candida albicans* is on the rise due to increasing numbers of immunosuppressed patients. The importance of the immune system in preventing overgrowth of the colonizing fungus and thereby limiting infection is well recognized and host protective mechanisms widely investigated. Only recently, it was recognized that the natural diversity in the fungal species could also influence the outcome of the interaction between the fungus and the host. *C. albicans* strain-specific differences are complex and their regulation at the genomic, genetic, and epigenetic level and by environmental factors is only partially understood. In this review, we provide an overview of the natural diversity of *C. albicans* and discuss how it impacts host-fungal interactions and thereby affects the balance between commensalism versus disease.

## 1. Introduction

*Candida albicans* is found as a commensal in the human oral, gastrointestinal, and reproductive tracts in a large proportion of healthy individuals, but it may become pathogenic when host defenses are breached or under condition of microbial dysbiosis. *C. albicans* disease symptoms range from mild to more severe superficial infection of the oral and vaginal mucosa, the skin, and the nails [1,2,3], affecting many millions of people worldwide [4]. More rarely, *C. albicans* reaches the bloodstream and disseminates to internal organs to cause systemic disease associated with high mortality rates [5]. While the integrity of epithelial barriers and a functional immune system are well known key determinants for protection against opportunistic *C. albicans* infections, it has become clear that variations in the fungal species contribute additional risk factors for disease development in susceptible hosts. Diversity in *C. albicans* can affect the morphology, the fungal behavior at the host interface, the growth rate, and resistance to stressors, such as antimicrobial peptides or antifungals. The transitions between different morphotypes (yeast, hyphae, pseudohyphae) and cell types (white, opaque, grey, gastrointestinally induced transition (GUT) cells) are reversible processes, mainly determined by the host environment and thus difficult to study in vitro.

Although variations between *C. albicans* natural isolates were noticed decades ago [6], most of the current understanding of *C. albicans*-host interactions is based on experimental studies with a limited number of strains, first and foremost the highly virulent strain SC5314. Only a few studies so far have examined the consequences of the fungal diversity on the outcome of infection. Most attempts to link specific genomic variations in *C. albicans* with phenotypic changes focused on drug resistance [7]. However, expression of virulence factors and the interaction with host cells also vary between genetic backgrounds of *C. albicans* and can greatly impact on the pathogenicity of the fungus during systemic and mucosal infections [8,9,10]. To provide an example, differences in the composition of the fungal cell wall and in the exposure of β-glucan between *C. albicans* isolates was found to determine the requirement for the fungal receptor Dectin-1 expressed primarily by myeloid immune cells during experimental systemic infection [11] and to influence the competitive fitness of the fungus in the murine gastrointestinal tract [12].

Together, these studies highlight the relevance of the intraspecies diversity of *C. albicans* for the outcome of the fungal-host interaction. However, the current knowledge about relevant fungal differences that explain the diverging behavior of natural *C. albicans* isolates in the host remains incomplete. In this review, we discuss the current understanding of the genetic, genomic, and phenotypic variations that have been described in *C. albicans* and how they modulate the outcome of the interplay with the host.

## 2. Host Responses to *C. albicans*

As a commensal, *C. albicans* is in constant interaction with the host epithelium. Progression from commensalism to infection implies numerous changes in the interplay between the fungus and the host, including enhanced adhesion, invasion, and induction of epithelial damage [13]. Epithelial cells can differentiate between more or less pathogenic forms of *C. albicans*, in response to which they activate distinct signaling pathways [14]. Recently a novel epithelial receptor, EphA2, required for maximum antifungal response was identified. It binds to β–glucan and thereby senses the epithelial fungal cell burden [15].

Tissue-invading fungi trigger the release of danger-associated molecules including alarmins such as interleukin-1α (IL-1α) and chemokines that promote the recruitment of neutrophils and inflammatory monocytes to the site of infection [16] where they contribute to antifungal defense [17,18]. Both the tissue resident and newly recruited myeloid cells of the innate immune system sense *C. albicans*. Recognition of fungal carbohydrates and other pathogen associated molecular patterns (PAMPs) in the fungal cell wall via pathogen recognition receptors (PRRs), in particular those of the C-type lectin receptor (CLR) family, leads to immune activation and the production of cytokines [19]. It can also promote the induction of adaptive immunity, including the generation of interleukin-17 (IL-17)-producing lymphocytes. IL-17 plays a unique role in protecting the mucosal epithelium from *C. albicans*, presumably by promoting the production of antimicrobial peptides [20,21]. Host defense against systemic candidiasis is dominated by the action of neutrophils and monocytes, which prevent fungal (over)growth and dissemination [22]. Neutrophils however can also drive immunopathology in certain settings [23,24]. Moreover, neutrophils are believed to promote significantly the development of disease symptoms during vulvovaginal candidiasis (VVC) [25].

The relative contribution of individual immune pathways to the host defense against *C. albicans* may vary in a strain-dependent manner. This was exemplified recently in the experimental mouse model of oropharyngeal candidiasis (OPC), where fungal strains were found to vary in their capacity to persist in the host [10]. Interestingly, the source of the isolates did not seem to correlate with pathogenicity [10]. This was also true when strains were assessed in different experimental models [26,27]. In the OPC model, a high degree of damage induction by *C. albicans* strains correlated with strong inflammatory response and rapid fungal clearance, while strains inducing low epithelial cell damage triggered only a low degree of inflammation and in consequence colonized the host persistently [10]. In contrast, IL-17 and its target genes were strongly induced by both high and low virulent strains indicating that the IL-17 pathway does not hinder fungal colonization, although it can prevent fungal overgrowth in both cases [10]. These findings show the importance of strain variations in modulating disease outcome. Still, more research is required to obtain a broader understanding of strain-specific versus conserved antifungal defense mechanisms in different host tissues. Also, it remains to be seen how these findings translate to the human host as all current data on how fungal diversity can impact the fungal-host interplay stem from experiments in vitro and in experimental mice, in which *C. albicans* is not a commensal.

## 3. Genetic and Genomic Variations

*C. albicans* is a highly successful organism, both as a commensal and a pathogen. This is thought to be—at least in part—due to the high degree of plasticity of its genome, which enables the fungus to grow in many different niches in the human host and to rapidly adapt to extreme stress conditions such as those imposed by the host immune system or by antifungal drugs [28]. Genomic variations are found in commensal isolates [29], but they may also be acquired and further enriched under stress conditions [30,31].

The diploid genome of *C. albicans* is approximately 14.4 megabases in size and organized in 8 chromosomes [32,33]. Based on multilocus sequence typing, the natural *C. albicans* population has been stratified in at least 18 phylogenetic groups (clades) [34]. Recent advances in whole genomic sequencing have further advanced the understanding of the genomic organization of *C. albicans* [9,35]. *C. albicans* populations isolated from the human host are mainly clonal when analyzed by multilocus sequence typing or similar approaches, suggesting that this diploid fungus reproduces predominantly clonally [36,37,38]. There is also evidence for low levels of DNA recombination in the population, possibly due to parasexual mating [39].

The genome is largely heterozygous, and heterozygosity is thought to be an important virulence trait of *C. albicans* [40]. Haploid forms of *C. albicans* have been isolated and found to be viable, but they show marked defects in fitness and virulence [41]. The observation that auto-diploids show the same fitness defects as haploids highlights the benefit of heterozygosity for the fungus [41]. Variations in chromosome organization and copy number can rapidly generate diversity and thereby allow adaptation to changes in the environment of the fungus [42].

Loss of heterozygosity (LOH) is a major mechanism to generate genetic diversity in populations of heterozygous organisms [43,44]. Passaging *C. albicans* through the murine host was found to enhance LOH, indicating that host signals such as oxidative stress are a main driver of the process [45]. LOH can also occur upon exposure to antifungal drugs [30] and other environmental stresses [46]. The adaptive benefits of LOH under selective pressure may result from alterations in gene expression levels or the selection of mutant alleles [47].

Aneuploidy, the loss or additional presence of one or more chromosomes, represents an alternative mechanism for how *C. albicans* can rapidly adapt to stressful environments. Aneuploidy of a specific chromosome can confer phenotypic changes by virtue of the copy number of specific genes on that chromosome relative to the copy number of other genes [28]. Although the presence of aneuploid chromosomes often carries a fitness cost, such as the reduction in growth rate, higher demands associated with DNA replication, and difficulty in segregating aneuploid chromosomes [48,49,50], aneuploidy can provide a selective advantage under stress conditions [42]. This has also been observed in other fungal pathogens such as *Cryptococcus neoformans* [44]. Several studies have documented how specific aneuploidies can directly confer drug-resistance in clinical isolates of *C. albicans* [51,52,53]. Aneuploidy also likely contributes to adaptation of *C. albicans* in the host as in vivo passaging of strains through a mouse was shown to induce aneuploid formation [45]. Because aneuploidy is often unstable when the fungus is kept under rich medium growth conditions, it may be rapidly lost after removal from the host niche and, thus, difficult to detect ex vivo [54].

In addition to LOH and aneuploidy, recombination between homologous chromosomes including gene conversion events can also impact on the phenotype of *C. albicans* [55]. This was described for instance during the parasexual cycle of *C. albicans* by concerted chromosome loss linked to recombination events [56]. Thereby, the reproductive cycle may also contribute to generating genomic variation in the fungus [57]. In conclusion, the remarkable genome plasticity observed in *C. albicans* represents a central mechanism of adaptive evolution promoting the fitness of the fungus, resistance to environmental stress, and interaction with the host.

Additional mechanisms of phenotypic diversification in *C. albicans* are achieved by sequence variations in coding or non-coding regions and at the transcriptional, translational, and post-translational level. The best studied examples of variations that affect the nucleotide sequence of individual genes are from drug resistance studies. There, mutations in genes encoding antifungal targets (e.g., *ERG11*, *FKS1*) and drug efflux pumps (e.g., *CDR1*, *CDR2*, *MDR1)* have been discovered and explain the progressive increase in resistance of isolates that have been exposed to antifungal treatments [58,59].

The expression of many *C. albicans* virulence genes is differentially regulated by environmental factors and/or in a cell-type specific manner (see below for hyphal-specific gene regulation). It is further influenced by differential chromatin states that are important in adaptation processes. Dynamic remodeling of telomeric heterochromatin occurred upon environmental changes [60]. The histone acetyl transferase Hat1 was found to mediate oxidative stress tolerance and resistance to azoles in *C. albicans* [61]. Moreover, DNA methylation in *C. albicans* was linked to morphological switching and iron metabolism, both processes important for virulence [62]. Therefore, epigenetic modulations add another level of plasticity, which may result in strain-specific differences.

Posttranscriptional regulatory mechanisms of genes involved in pathogenesis and morphology have also been described in *C. albicans,* including the regulation of mRNA stability and localization [63,64].

At the translational level, phenotypic variation is enhanced by the atypical codon usage at CUG sites incorporating serine instead of leucine and a remarkable codon assignment flexibility [65,66]. This adaptive mistranslation creates genomic alterations that can affect fungal growth and fitness in particular under selective pressure as exemplified by fluconazole administration [67].

An additional posttranslational mechanism, with impact on *C. albicans* morphogenesis, virulence and drug resistance is mediated by the acetylation of the heat-shock family protein Hsp90 thereby promoting its function as a stress response regulator [68,69]. Some virulence factors are also regulated at the posttranslational level through enzymes such as aspartic proteases or phospholipases that modulate the function of their targets under specific environmental conditions [70,71].

Together, these examples highlight the plethora of mechanisms that *C. albicans* uses to increase its adaptive flexibility in response to environmental cues, host factors, and antifungal stressors.

## 4. Morphotype Variations

*C. albicans* exist in distinct morphological forms, including yeast, hyphae, and pseudohyphae. They differ in size, shape, cellularity, and in their mode of division. While unicellular spherical yeast cells reproduce by budding, tube-shaped hyphal cells remain firmly attached one to another to produce multicellular filamentous structures [72]. Ellipsoid-shaped pseudohyphal cells have features of both yeasts and hyphae. They consist of strings of elongated yeast cells that fail to separate, but in contrast to hyphae, they have constrictions at the sites of septation and are wider than hyphae [72,73]. Diverse environmental and host conditions promote the reversible transition between the distinct morphotypes including contact of *C. albicans* to body surfaces, exposure to host cell molecules and/or changes in oxygen availability and carbon sources [74], or influences from the microbiota and their metabolites [75]. These host conditions can at least in part be simulated by elevated temperature (37 °C), adhesion to inert surfaces, or the presence of serum to promote morphotype switching in vitro [72]. 

In addition to environmental cues that regulate the balance between different *C. albicans* morphotypes, the genetic variation in the species determines the propensity of individual strains to form hyphae or pseudohyphae. Changes in the sequence and/or expression of cell type-specific genes can greatly impact the morphology of a strain and thereby the functional diversity that is observed within the species overall.

Hyphal morphogenesis is coupled to virulence. This is best characterized for yeast and hyphae, while the role of pseudohyphae is less well understood. Yeast cells are generally viewed as the commensal form colonizing the mucosal epithelia, while hyphae are the more pathogenic form, promoting epithelial invasion that leads to disease symptoms [76]. In the context of systemic infections, yeast cells facilitate dissemination of the fungus through the blood stream, whereas the transition into the filamentous form is a prerequisite for induction of pathology by the fungus [77]. Mutants unable to switch between the yeast and hyphal form are compromised in virulence [78,79]. This is linked to a different capacity of the individual *C. albicans* morphological forms to interact with and invade the host.

Major changes occur in the structure and composition of the fungal cell wall upon morphotype switching [76]. This entails alterations in the interaction of *C. albicans* with host cells via CLRs and other PRRs and in the ensuing innate and adaptive antifungal immune response [80]. Moreover, yeast and hyphal cells display important differences in their capacity to adhere, invade, and cause damage to epithelial cell barriers. The fungal molecules that mediate these processes are under the control of the hyphal gene expression program [81]. These include adhesins (e.g., Hwp1 and Als3), invasins (e.g., Als3), and tissue-degrading enzymes such as secreted aspartyl proteinases and phospholipases [82,83,84]. Likewise, the gene encoding a recently identified cytolytic peptide toxin Ece1, a key fungal determinant responsible for the induction of host cell damage [85], is selectively expressed during hyphal growth. However, Ece1 is not essential for filamentation as an *ECE1*-deletion mutant has no defect in hyphae formation [85], underlining that filamentation and damage induction are two separate processes. The capacity to induce epithelial damage greatly varies between natural *C. albicans* isolates [10]. The role of Ece1 in damage induction may be strain specific as no correlation between Ece1 expression and the level of damage induction was found so far across a number of natural isolates [10]. In the murine OPC model, differences in epithelial damage translate into variable degrees of inflammation and immune cell recruitment to the infected tissue and, in turn, result in either clearance or persistence of the fungus in the oral mucosa [10].

Besides yeast, hyphae, and pseudohyphae, additional much rarer growth forms of *C. albicans* have been described. For instance, the production of chlamydospores [86] or highly resistant persister cells [87] were observed under environmental conditions characterized by low nutrient availability or elevated stress. Moreover, some strains of *C. albicans* can show flake-like growth. In these strains, budding yeast cells stay attached and do not separate from the mother cell, resulting in yeast cell aggregates. Mutations in the promotor *FLO1* and the transcription factor Ace2 were found to cause this aggregative growth in *C. albicans* [88,89]. Interestingly, flake-like multicellular yeast growth creates a bottleneck for evolution and, thus, might be beneficial for adaptation to a host environment, as clusters of genetically identical cells (not single cells) are exposed to selective pressures and thus enable faster adaptation [90].

Mixed populations of *C. albicans* yeast, hyphae, and pseudohyphae are observed in biofilms [91,92]. These densely packed communities of cells encased in extracellular matrix preferentially grow on mucosal surfaces or solid substrates such as catheters. They constitute a major virulence attribute of *C. albicans* because of their high intrinsic resistance to antibiotics and the mammalian immune system. Biofilm formation may also vary between strains [93].

From an evolutionary perspective, populations with high morphological variability may be better equipped to adapt to fluctuating environmental conditions. Selective pressure will enable survival and propagation of the cells with the highest fitness and, subsequently, lead to adaptation of the strain. This may be of clinical relevance as versatile fungal strains will be able to better persist in the presence of host defenses or antifungals.

## 5. Niche-Specific Morphology Switching

In addition to the classical ‘white’ yeast cells described above, *C. albicans* can adopt several additional yeast cell-like morphologies [91]. Opaque, grey, and GUT cells have an elongated shape compared to their round-to-oval shaped white counterpart and they exhibit distinct properties. While white cells survive and thrive in a variety of different environments, opaque, grey, and GUT cells exhibit enhanced fitness only in specific host niches. Their preference for specific host niches is reflected in their metabolic program. While white cells display a transcriptional signature of using fermentation pathways and glycolysis, opaque cells preferentially use pathways involved in oxidative respiration [94]. They were shown to have a high fitness in a neonatal mouse skin colonization model [95]. Grey cells are selectively induced by exposure to nutrient-rich growth medium and display a particularly high growth rate in an ex vivo tongue infection model [94]. GUT cells in contrast display the highest fitness in the gastrointestinal (GI) tract but not in other environments of experimentally colonized mice [96]. Thus, a common feature of these newly described elongated yeast-like phenotypes may be their specialization for commensalism.

Switching between the different yeast-like cell types is a transient and reversible process that strictly depends on environmental factors. White-opaque switching, although occurring stochastically and at low frequency, can be promoted by *N*-acetylglucosamine, hypercarbia, and an acidic pH [97,98,99,100]. While it was long thought that white cells, which are usually heterozygous (a/α) at the mating type locus (MTL), need first to undergo homozygosis to a/a or α/α to become switching-competent, it is now clear that heterozygous a/α cells are also able to switch to the opaque state [95]. White-to-opaque switching is under the control of the master regulators Wor1 and Wor2 [97,101] that act in a circuit of transcriptional regulators which further include the recently identified regulator Wor4 [102] and the transcriptional repressor Ssn6 [103]. In addition to transcriptional regulation, epigenetic mechanisms, e.g., histone deacetylation, were also found to regulate white-opaque switching [104]. Treatment of *C. albicans* with a histone deacetylase inhibitor or selective genetic targeting of the histone deacetylase *HDA1* increased switching from the white to the opaque state [105]. The combined use of antifungals with histone deacetylase inhibitors, to reduce fungal adaptation towards drug resistance, might thus represent a promising therapeutic strategy [106].

The capacity of *C. albicans* to switch between the white and opaque state is a prerequisite for the fungus to mate. Only opaque cells that are haploid at the mating type locus are mating competent [107]. Mating occurs between two opaque cells with opposite mating type that fuse to produce diploid a/α cells followed by chromosome loss in tetraploid cells and possibly genomic recombination [108]. Thereby, this parasexual circle creates phenotypic diversity in *C. albicans* with an impact on host virulence and resistance [57]. Besides the link to mating, which is rare in *C. albicans*, white-opaque switching may represent an immune evasion strategy. In contrast to their white counterparts, opaque cells are more resistant to phagocytosis by host cells [109,110,111] and do not release chemoattractants for polymorphonuclear leukocytes [112]. Opaque cells have also been shown to display an altered sensitivity to at least some antifungal drugs [113]. Under specific conditions, opaque cells can form hyphae similarly to white cells, although the filamentation program largely differs between the two cell types [114]. Opaque cells have been shown to filament also in vivo to establish a lethal infection, at least at low temperature [111].

Although it has become clear that the phenotypic plasticity contributes to *C. albicans*’ ability to colonize and infect different host niches, more research is needed to gain a comprehensive view of the regulation of different cell types in vivo in different host niches and how they impact the fungus-host interaction.

## 6. Conclusions

Although impaired immune functions and microbial dysbiosis in the host remain the primary determinants of opportunistic *C. albicans* infections, variations within the fungal species significantly contribute to the development and severity of disease symptoms. Diversity exists at multiple levels ranging from nucleotide to morphological differences. Heterogeneity at the genomic, cellular, or population level facilitates adaptation to environmental changes and enhances the competitive fitness of the fungus in diverse host niches.

Understanding the complex regulation of the phenotypic and functional variations in *C. albicans* remains a major challenge. While several studies have explored how functionally distinct strains affect the interaction with the host, the molecular basis for the observed phenotypic differences remains unclear in most cases. The field is awaiting identification of specific genetic and genomic variations that translate into relevant phenotypic changes in *C. albicans* to affect the balance between commensalism and pathogenicity in different host niches.

Studying the diversity of *C. albicans* is complicated by the transient and reversible nature of many of the fungal variations, including the diverse morphotypes as well as their strict dependence on host environmental factors. They may not remain stable once removed from the host context. Moreover, the lack of standardized protocols for studying the niche-specific cell types hampers the comparison of results from different laboratories.

Key open questions include how variations in the host and in the microbiota can create diversity in the fungal species and how they may promote the switch in *C. albicans* from commensalism to pathogenicity. While many examples exist for how *C. albicans* adapts to antifungals, there is a current gap in knowledge on how the immune system and the microbiota modulate fungal genetics and genomics during commensalism and pathogenicity. A better understanding of these processes will contribute to the development of novel and possibly personalized strategies to predict and improve patient outcome.
